# Infiltrating mast cells increase prostate cancer chemotherapy and radiotherapy resistances *via* modulation of p38/p53/p21 and ATM signals

**DOI:** 10.18632/oncotarget.6372

**Published:** 2015-11-16

**Authors:** Hongjun Xie, Chong Li, Qiang Dang, Luke S. Chang, Lei Li

**Affiliations:** ^1^ Chawnshang Chang Sex Hormone Research Center, Department of Urology, The First Affiliated Hospital, Xi'an Jiaotong University, Xi'an 710061, China; ^2^ CAS Key Laboratory of Infection and Immunity, Institute of Biophysics, Chinese Academy of Science, Beijing 100101, China

**Keywords:** prostate cancer, docetaxel, radiotherapy, p38, ATM

## Abstract

Early studies indicated that mast cells in prostate tumor microenvironment might influence prostate cancer (PCa) progression. Their impacts to PCa therapy, however, remained unclear. Here we found PCa could recruit more mast cells than normal prostate epithelial cells then alter PCa chemotherapy and radiotherapy sensitivity, leading to PCa more resistant to these therapies. Mechanism dissection revealed that infiltrated mast cells could increase p21 expression *via* modulation of p38/p53 signals, and interrupting p38-p53 signals via siRNAs of p53 or p21 could reverse mast cell-induced docetaxel chemotherapy resistance of PCa. Furthermore, recruited mast cells could also increase the phosphorylation of ATM at ser-1981 site, and inhibition of ATM activity could reverse mast cell-induced radiotherapy resistance. The *in vivo* mouse model with xenografted PCa C4-2 cells co-cultured with mast cells also confirmed that mast cells could increase PCa chemotherapy resistance *via* activating p38/p53/p21 signaling. Together, our results provide a new mechanism showing infiltrated mast cells could alter PCa chemotherapy and radiotherapy sensitivity *via* modulating the p38/p53/p21 signaling and phosphorylation of ATM. Targeting this newly identified signaling may help us better suppress PCa chemotherapy and radiotherapy resistance.

## INTRODUCTION

Prostate cancer (PCa) is the second most common diagnosed cancer worldwide, and the most common cancer in men of developed countries [1]. It is well known that androgen- androgen receptor (AR) signals play key roles in PCa progression. Androgen Deprivation Therapy (ADT) to reduce or prevent androgens binding to AR is the major treatment for the advanced PCa. However, it will eventually relapse and develop into castration resistance after 1–2 years of treatment [2].

More evidence suggested that PCa progression might be associated with inflammatory cells infiltration. PCa treated with ADT may result in recruitment of various immune cells, including T cells, dendritic cells, natural killer cells, mast cells, macrophages and neutrophils to prostate tumor microenvironment [3–5]. However, the impacts of these infiltrating immune cells together with the inflammatory cytokines they secrete on PCa progression and therapies remain unclear.

Mast cells have been reported to play important roles in allergy or angiogenesis [6, 7]. In PCa, mast cells increase during development of PIN in TRAMP mice and human tissue [8]. Our early results showed that mast cells could enhance PCa cell invasion via increasing stem/progenitor cell population [9]. ADT with enzalutamide could increase PCa neuroendocrine (NE) differentiation capabilities via recruitment of infiltrating mast cells [10].

Chemotherapy with docetaxel has been proved to be able to improve survival of PCa patients at castration resistant stage [11, 12]. However, the chemo-resistance may develop rapidly without clear mechanism [13, 14] and its linkage to inflammation also remains unclear. Similarly, though radiotherapy (RT) for localized PCa also contribute to improved survival of patients [15], the influences of immune responses on RT remain to be further elucidated [16, 17].

Here we found infiltrating mast cells could enhance PCa resistance to chemotherapy and radiotherapy *via* activation of p38/p53/p21 and ATM signals.

## RESULTS

### Prostate cancer recruits more mast cells than normal prostate

**Figure 1:**
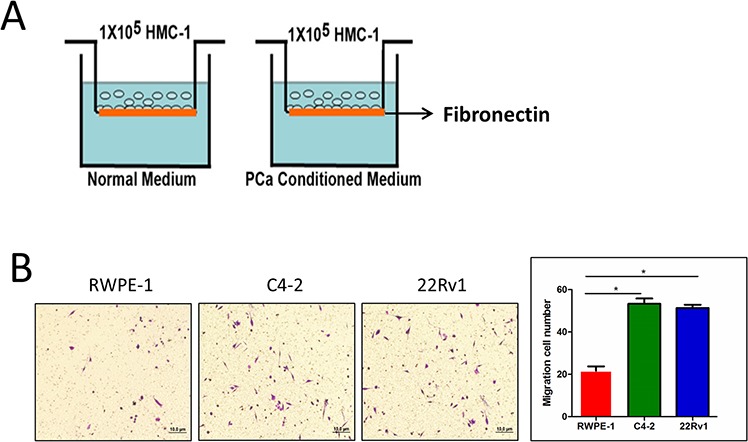
Prostate cancer recruits more mast cells than normal prostate **A.** Cartoon illustration of the mast cell migration assay. The insert upper wells were pre-coated by 10 ng/ml fibronectin. HMC-1 cells (mast cells, 1 × 10^5^) were placed in the upper chamber and the conditioned medium was placed in the bottom wells to assay the migration of mast cells. After 4 hrs, the bottom sites of insert wells were fixed and stained to visualize the migrated mast cells. **B.** PCa cells promote mast cell migration. Mast cells (1 × 10^5^) were added in the upper well, we placed non-malignant prostate RWPE-1 cell conditioned medium and PCa C4-2 and CWR22Rv1 (22Rv1) cells conditioned medium to do migration assay. The right panel is the quantitative data for migrated mast cells. Results were presented as the average values and represented as mean± SEM. *p < 0.05.

Previous studies suggested that several tumors, including PCa, might be able to recruit mast cells [9, 10, 18]. Using the Boyden chamber migration system (see the cartoon in Figure 1A), we found here that PCa C4-2 cells have better capacity than normal prostate RWPE-1 cells to recruit more mast cells (Figure 1B). Similar results were also obtained when we replaced C4-2 PCa cells with PCa CWR22Rv1 cells (Figure 1B).

Together, results from Figure 1A–1B suggest that PCa may have better capacity than normal prostate to recruit mast cells.

### Recruited mast cells alter the PCa chemotherapy sensitivity

**Figure 2:**
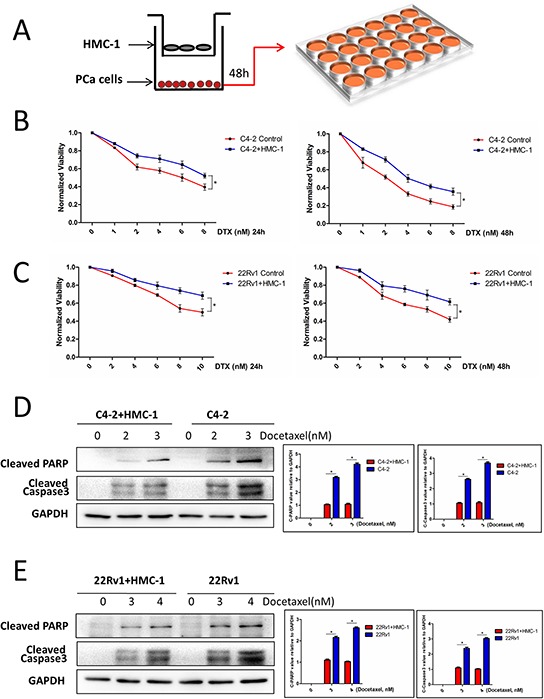
PCa cells co-cultured with mast cells show chemotherapy resistance **A.** The cartoon illustrates the co-culture system. We co-cultured PCa cells with mast cell for 2 days, then the trypsinized PCa cells were seeded in 24-well plates, after adherence, treated with different doses of docetaxel for 24 and 48 hrs, tested with MTT. Consider absorbance of 0 nM as control, all absorbance of other dose was compared with control. **B.** C4-2 cells co-cultured with mast cells showed more resistant to docetaxel treatment. *p < 0.05. **C.** CWR22Rv1 (22Rv1) cells co-cultured with mast cells showed more resistant to docetaxel treatment. *p < 0.05. **D.** C4-2 cells co-cultured with mast cells showed less expression of cleaved PARP and cleaved Caspase3 with docetaxel treatment, the right panel is the quantitative data. *p < 0.05. **E.** CWR22Rv1 (22Rv1) cells co-cultured with mast cells showed less expression of cleaved PARP and cleaved Caspase3 with docetaxel treatment, the right panel is the quantitative data. *p < 0.05.

To study the potential consequences of PCa cells to recruit more mast cells, we then applied the co-culture system to assay the chemo-sensitivity of PCa under docetaxel treatment (Figure 2A), and results revealed that after recruitment of more mast cells, the PCa C4-2 cells became more resistant to docetaxel chemotherapy of both 24 and 48 hours (Figure 2B). Similar results were also obtained when we replaced PCa C4-2 cells with CWR22Rv1 cells (Figure 2C).

Interestingly, we also found that recruited mast cells could inhibit docetaxel-induced cell apoptosis in C4-2 and CWR22Rv1 cells with decreased apoptosis marker of cleaved PARP and cleaved caspase3 expression (Figure 2D–2E).

Together, results from Figure 2A–2E suggest that infiltrating mast cells could decrease docetaxel-induced PCa cell apoptosis and enhance PCa cells’ resistance to docetaxel.

### Mechanism why recruited mast cells could alter PCa cells chemotherapy sensitivity

**Figure 3:**
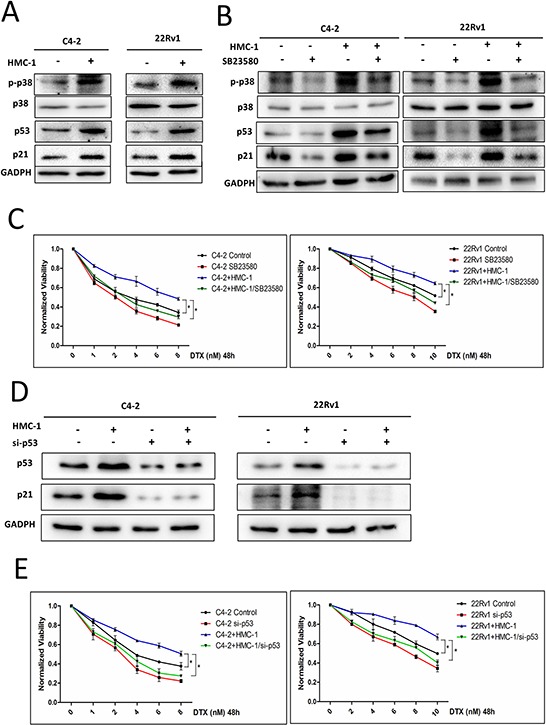

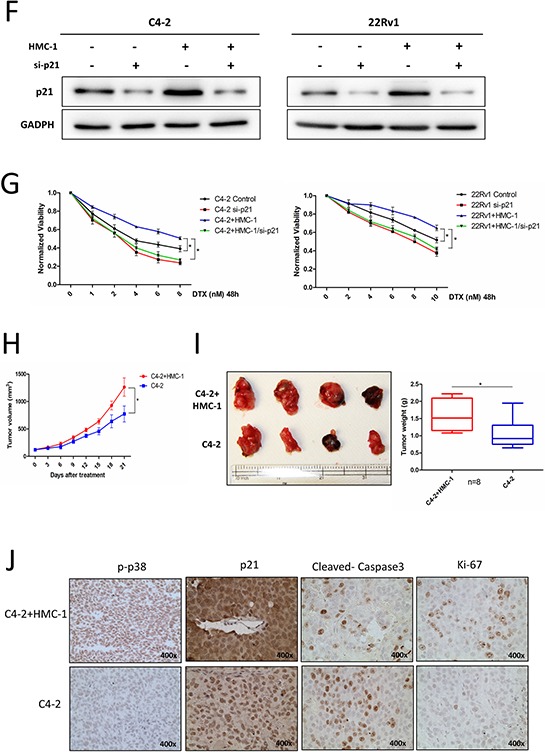
Mechanism why recruited mast cells can alter PCa cell chemotherapy sensitivity and *in vivo* data **A.** PCa C4-2 and CWR22Rv1 (22Rv1) cells show increased expression of p-p38, p53 and p21 after co-culture with mast cells. **B.** Targeting p38 with inhibitor SB23580 can decrease expression of p-p38, p53 and p21. **C.** Targeting p38 with inhibitor SB23580 can interrupt mast cells induced docetaxel resistance. **D.** Knocking down p53 in PCa C4-2 and CWR22Rv1 (22Rv1) cells with and without co-culture with mast cells. **E.** Knocking down p53 in C4-2 and CWR22Rv1 (22Rv1) cells can reverse co-culture induced docetaxel resistance. **F.** Knocking down p21 in PCa C4-2 and CWR22Rv1 (22Rv1) cells with and without co-culture with mast cells. **G.** Knocking down p21 in C4-2 and CWR22Rv1 (22Rv1) cells can reverse co-culture induced docetaxel resistance. **H.** The growth curve of tumors in these two groups after treatment of docetaxel. **I.** Left, the representative figure for volume of subcutaneously xenografted tumors treated with docetaxel. Right, the quantitative data for the tumor weight. *p < 0.05. **J.** IHC staining for p-p38, p21,cleaved caspase3 and ki-67 in mice tumor tissues.

To dissect the molecular mechanism how recruited mast cells could alter PCa chemotherapy sensitivity, we focused on the p38-p53-p21 signals since early studies indicated that they might play key roles in altering chemotherapy sensitivity [19]. As shown in Figure 3A, the expression of phosphorylation-p38 (p-p38), p53 and p21 were increased in PCa C4-2 and CWR22Rv1 cells after co-culture with mast cells (Figure 3A). Furthermore, we also found that the expression of phosphorylation-p38 (p-p38), p53 and p21 were increased even in the presence of DTX (Supplementary Figure S1A).

We then applied the interruption approach with the inhibitor of p38 (SB23580) to suppress phosphorylation of p38. Results showed that inhibition of p38 signaling could partially reverse the mast cell-induced expression of p-p38, p53 and p21, with partially restoration of PCa cells sensitivity to docetaxel treatment (Figure 3B–3C). When we knocked down p38, we also obtained the similar results (Supplementary Figure S1B). Furthermore, knocking down p53 or p21 could also partially reverse mast cell-induced PCa docetaxel resistance (Figure 3D–3G).

Together, results from Figure 3A–3G and Supplementary Figure S1A–S1B suggested that infiltrating mast cells could induce PCa cells resistance to docetaxel *via* activating p38/p53/p21 signaling.

### Mast cells enhance PCa cells chemotherapy resistance *in vivo*

To demonstrate the *in vitro* cell lines results above in the *in vivo* mouse model, we subcutaneously injected PCa cells into 6 to 8 week old male nude mice. 8 mice were injected subcutaneously with 1 × 10^6^ C4-2 cells pre-co-cultured with mast cells for 1 week, as a mixture with Matrigel, 1:1 and another 8 mice were injected with 1 × 10^6^ C4-2 cells, as a mixture with Matrigel, 1:1. After 2 weeks, the mice were then treated with docetaxel (15 mg/kg, 2 times/week) for another 3 weeks before sacrifice.

The results, after continue monitoring the growth curve of these two groups mice, revealed that mice pre-treated with mast cells showed more resistance to docetaxel (Figure 3H), with bigger tumor volume and heavier tumor weight than those in the control group (Figure 3I). Results from IHC staining of p-p38 and p21 were also in agreement with *in-vitro* co-culture studies, showing that infiltrating mast cells could increase p-p38, p21 and ki-67 expression, but decrease cleaved caspase3 expression (Figure 3J).

### Recruited mast cells alter the radiotherapy sensitivity

**Figure 4:**
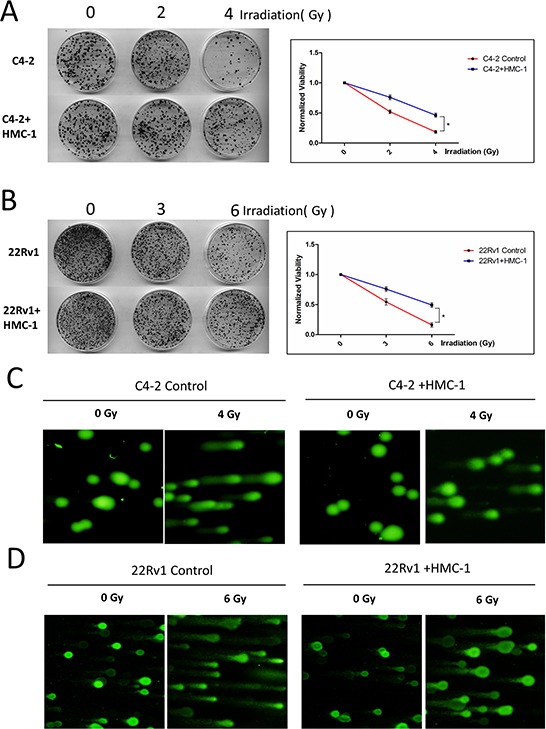
PCa cells co-cultured with mast cells show radiation resistance **A.** Colony formation assay. Seed 1000 co-cultured C4-2 cells and naive C4-2 cells into 6 cm dishes respectively, after adherence, treat cells with 2 and 4 Gy γ-radiation for one time and culture them for two weeks, every 3–4 days to change fresh medium, fix and staining. The right panel is the quantitative data for colony formation assay. *p < 0.05. **B.** Seed 1000 co-cultured CWR22Rv1 (22Rv1) cells and naive CWR22Rv1 (22Rv1) cells into 6 cm dishes respectively, after adherence, treat cells with 3 and 6Gy γ-radiation for one time and culture them for two weeks, every 3–4 days to change fresh medium, fix and staining. The right panel is the quantitative data for colony formation assay. *p < 0.05. **C.** Alkaline Comet assay of C4-2 cells with or without co-culture with mast cells after 4Gy of IR, showing decreased DNA damage in the co-culture group. **D.** Alkaline Comet assay of CWR22Rv1 (22Rv1) cells with or without co-culture with mast cells after 6Gy of IR, showing decreased DNA damage in the co-culture group.

In addition to altering the chemotherapy sensitivity, we are also interested to see the effect of recruited mast cells on the resistance of PCa to radiotherapy. Using colony formation assay, we found C4-2 cells alone had better sensitivity to radiotherapy than those co-cultured with mast cells (Figure 4A). Similar results were also obtained when we replaced C4-2 PCa cells with CWR22Rv1 cells (Figure 4B).

Using another approach to measure the radiation sensitivity with the alkaline-comet assay, we found the PCa cells (C4-2 and CWR22Rv1) co-cultured with mast cells had less DNA damage after radiotherapy compared with the control group (Figure 4C–4D).

Together, results from Figure 4A–4D suggested that recruited mast cells could also enhance PCa cells’ resistance to radiotherapy.

### Mechanism dissection why recruited mast cells could alter radiotherapy sensitivity

**Figure 5:**
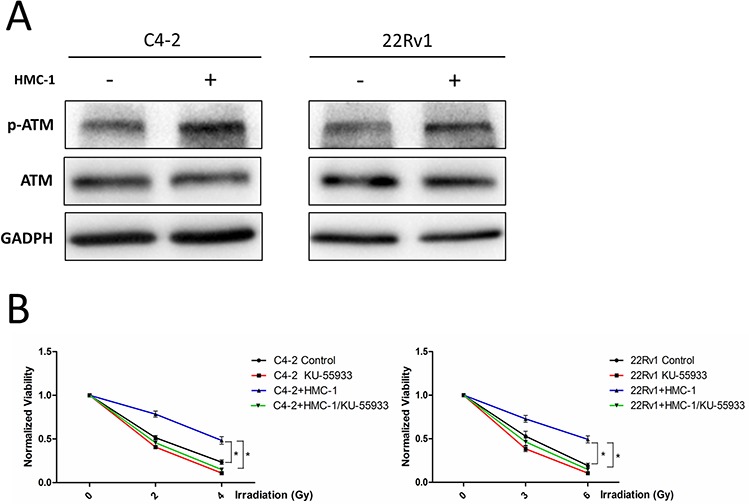
Mechanism why recruited mast cells can alter radiotherapy sensitivity **A.** C4-2 and CWR22Rv1 (22Rv1) cells show increased expression of phosphorylation of ATM at ser-1981 site after co-culture with mast cells. **B.** ATM kinase inhibitor KU-55933 (10 uM) could reverse co-culture induced PCa cell radiation resistance.

To dissect the molecular mechanism how recruited mast cells alter PCa radiotherapy sensitivity, we focused on the expression of ATM, the key player in response to radiation [20, 21]. We found that the expression of phosphorylated ATM (ser-1981) was increased after co-culture with mast cells in C4-2 and CWR22Rv1 cells (Figure 5A), but the activity of another key molecule ATR had no change (Supplementary Figure S2A).

We then applied the interruption approach with ATM kinase inhibitor KU-55933 to see if inhibition of ATM activity could abolish co-culture-induced radiotherapy resistance of PCa cells. As shown in Figure 5B, addition of ATM kinase inhibitor could reverse co-culture-induced radiotherapy resistance of PCa cells.

## DISCUSSION

Docetaxel is a well-established anti-microtubule chemotherapy agent. Microtubules are dynamic filamentous proteins that play key roles in a range of cellular functions providing integrity and preserving cellular architecture and cellular protein transport [22, 23]. Docetaxel-based chemotherapy showed some improvement of approximately 3 months in median overall survival in PCa when compared with mitoxantrone treatment [24]. However, most patients with docetaxel chemotherapy still suffered from progression of their disease within 1 year from the start of treatment [25], suggesting that there exist some molecular mechanisms that lead to the development of docetaxel resistance.

Early mechanism dissection suggested that docetaxel resistance might be linked to altering the AR signals including AR gene amplification, AR mutations, and overexpression of AR co-regulators [26]. Other AR-independent mechanisms involved the modulating the Akt/PI3K and MAPK/ERK [27, 28], mTOR [29], nuclear factor-kappa B (NFκB)/IL-6 [30] and Hedgehog [31] signaling pathways. Furthermore, the interactions between cancer cells and the surrounding microenvironment [32, 33], as well as their secreted cytokines and growth factors (for example, IL-6 and stromal cell-derived factor 1), and altering the ECM [34] may also play key roles to development of docetaxel resistance.

Our results indicated that infiltrating mast cells could increase phosphorylation of p38 and interruption of this increased p38 could reverse mastcell-induced docetaxel resistance. This is interesting since P38 has been shown to play a dual role in progression of different cells: functions asmediators of apoptosis in some selective cells including neurons [35, 36] or cardiac cells [37, 38], yet also function as pro-tumorigenic role with positive correlation to bad prognosis in cancers. For example, p38 may lead to survival or proliferation of some cancers, including breast cancer [39], colorectal cancer [40], prostate cancer [41]. Here we found that infiltrating mast cells could increase phosphorylation of p38 and that inhibition of p38 could reverse mastcell-induced docetaxel resistance.

The tumor suppressor *p53* has an essential role in promoting antitumor drug response in its wild-type state, and tumor cells harboring wild-type *p53* are generally recognized as being more sensitive to antitumor agents [42], which is consistent with the concept that activation of wild type p53 is sufficient to induce cell death [43].

Unfortunately, such tumor cells do eventually become resistant to therapy [44, 45], and mutant or p53-null tumor cells undergo apoptosis are also involved in the development of chemotherapy resistance [46, 47]. For example, overexpression of wild-typep53 may be linked to the increased resistance to cisplatin-based chemotherapy in breast and ovarian cancer [48, 49], as well as in docetaxel resistancein PCa cells [19, 50].

Here we found that recruitment of mast cells to PCa cellsinduced docetaxel resistance via increased expression of wild-type p53. Although it has also been reported that DU145 (mutant p53) and PC3 (p53 null) cells were less sensitive than LNCaP and C4-2 cells expressing functional p53 in response to docetaxel, The reason for the conflict among these conclusions remains unclear while a possile explanation lies in the different passages or variants of prostate cancer cells used by different laboratories.

P21, the downstream gene of p53, functions as a regulator of cell cycle progression by binding to CDK/cyclin complexes, is associated with testicular cancer andovarian cancer resistance to cisplatin chemotherapy [51–53], and itsincreased expression is also associated with PCa cells docetaxel resistance [19, 54]. Here we found that infiltrating mast cells in PCa could also lead to increased p21 expression and its cytoplasmic accumulation *via* modulation of p38 signal, and that knocking down p21 could reverse mast cell-induced doxetaxel resistance.

Currently, radiationtherapy is one of the most common definitive treatment options for localized prostate cancer, and recent advances in volumetric based intensity modulated radiation therapy (IMRT) and image guided radiation therapy (IGRT) have permitted radiation dose escalation beyond 75Gy with external therapy, which has reduced both biochemical failure rate and the development of metastasis [55, 56]. However, a significant number of patients undergoing radiation therapy will develop locally persistent/recurrent tumors [57]. One possible reason for these failures is that there exists a subpopulation of prostate tumor cells with intrinsic radioresistance within the tumor. The ATM protein kinase is a key component of the signal transduction pathway activated byDNA damage [20, 21], and adiation can induce rapid intermolecular autophosphorylation which leads to dimer dissociation and ATM kinase activation [58]. The activated ATM protein kinase may then co-ordinate DNA damage response to alter the cell cycle checkpointsand DNA repair (e.g., p53) system [58, 59]. Cells lacking functional ATM protein show increased sensitivity to ionizing radiation [60]. Here we also found that infiltrating mast cells in PCa could induce PCa radiation resistance *via* activation of ATM signals. It has also been reported that ATM could activate p53 in response to DNA damage [59, 61, 62]. We found mast cells could increase ATM phosphorylation and p53 expression, but when we applied the ATM kinase inhibitor KU-55933, it failed to abrogate the mast-cell-induced increased expression of p53, which implied that increased expression of p53 induced by mast cells is due to the activation of p38 signal instead of ATM activation.

Mast cells play a key role in the pathogenesis of cardiovascular diseasesand cancers via secreting a variety of cytokines includingTNF-α, IL-3, IL-4, IL-5, IL-6, IL-10, IL-13, IL-14 and IL-16 [63–65]. We screened some candidates potentially activating p38 signal including IL-4, IL-5 and IL-6 [66–68], and results revealed that IL-6 increased in the medium of both C4-2 and CWR22Rv1 cells after co-culture with mast cells (Supplementary Figure S3). Interestingly, it is reported that increased expression of IL-6 could also activate ATM *via* increasing its phosphorylation [69]. Based on these results and reports, mast cell-induced activation of p38 and ATM signals may go through increased expression of IL-6 after co-culture with PCa cells.

**Figure 6:**
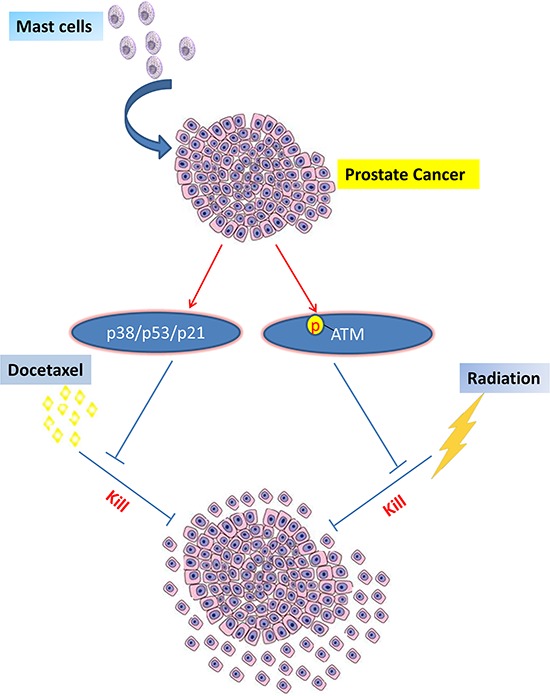
Mechanisms and regulatory pathways of mast cells promoted PCa docetaxel and radiation resistance Mast cells could enhance PCa cells docetaxel and radiation resistance via activation of p38/p53/p21 and ATM signals.

In summary, infiltrating mast cells can promote PCa chemotherapy and radiotherapy resistance *via* activating p38/p53/p21 and ATM signals (Figure 6). Future studies to target these newly identified signals may provide us with a new potential therapeutic approach to better battle PCa chemotherapy and radiotherapy resistance.

## MATERIALS AND METHODS

### Cell lines

C4-2 was gift from Dr. Jer-Tsong Hsieh of university of southwestern medical center and grown in RPMI with 10% fetal bovine serum. RWPE-1 and CWR22Rv1 cell line were purchased from the American Type Culture Collection (ATCC, Manassas, VA). RWPE-1 was grown in K-SFM media (Invitrogen, Grand Island, NY), CWR22Rv1 was grown in RPMI (Invitrogen). Human mast cell line HMC-1 was a gift from Dr. John Frelinger of eye institute of University of Rochester. HMC-1 was cultured in Iscove's modified Dulbecco's medium (IMDM) supplemented with 10% heat inactivated fetal bovine serum (FBS), 2 mM L-glutamine,100 IU/mL penicillin, 50 μg/mL streptomycin.

### Reagents and materials

GAPDH (6c5) and p53 (sc-126) antibodies were purchased from Santa Cruz Biotechnology(PasoRobles, CA). Cleaved PARP (5625), p21(#2947), Cleaved Caspase3(5A1E), p-P38 (#4511), p38 (D13E1), ATM (D2E2) and Phospho-ATM (Ser1981) antibodies were purchased from Cell signal Technology company (Boston, MA). ATM kinase inhibitor KU-55933 was purchased from Sigma-Aldrich Co. LLCcompany (St. Louis, MO). Docetaxel was purchased from LC Laboratories (Woburn, MA).

### Mast cell recruitment assay

Mast cell migration was detected by using a 24-well transwell assay. Briefly, prostate cells conditioned mediums were placed in the lower chamber of a 24-well. Mast cells (1 × 10^5^ cells) were then seeded in the upper chamber. The upper and lower chamber were separated by an 8 μm polycarbonate filter coated with fibronectin (10 μg/ml, sc-29011 Santa Cruz) and dried for 1 hr in the hood. The chambers were incubated for 4 hours at 37°C, Filters were then scraped, washed, fixed with cold methanol, and stained with 1% toludine blue. Cell migration was measured by counting the number of cells attached to the lower surface of the filter. Each conditioned medium was tested in triplicate. The results were expressed as the average of the number of migrating cells.

### Long term effect colony assay

PCa cells were cultured with or without mast cells for 48 hrs. PCa cells were plated in 60-mm culture dishes at densities of 1000 cells per plate and allowed to attach overnight, then cells were irradiated. The cell dishes were directly placed in the Cs^137^ irradiator, and the cells were irradiated at different doses treatments. Change fresh medium 2 hours later after radiation. Cells were incubated for 10 to 14 days after irradiation and then fixed with 10% methanol/10% acetic acid and stained with 0.1% crystal violet. Colonies containing more than 50 cells were counted. The plating efficiencies were determined for each treatment and normalized to controls. The curves were fitted using a second order polynomial function. The average normalized surviving fraction from three independent experiments and the S.E.M. were reported.

### Cell proliferation assay (MTT assay)

PCa cells cultured with or without mast cells for 48 hrs were plated into 24-well plates at a density of 5000 cells per well, treated with indicated doses of docetaxel. Collected the cells and did MTT assay after 24 hrs and 48 hrs: Add 250μl of 5 mg/ml MTT to each well. Incubate for 2 hours in incubator at 37°C. Remove media and add 150 μl DMSO. Cover with tinfoil and agitate cells on orbital shaker for 15 min. Read absorbance at 570 nm.

### Alkaline comet assay

PCa cells were co-cultured with mast cells and irradiated in described conditions. DNA lesions, including total base damage, DSBs and SSBs, were assessed using single-cell gel electrophoretic comet assays under alkaline condition (TREVIGEN, Gaithersburg, MD), the procedure was done according to the manufacturer's instructions. Slides were stained with SYBR Gold and visualized using a fluorescence microscope.

### Western blot analysis

Cells were lysed in RIPA buffer and proteins (20 μg) were separated on 10–12% SDS/PAGE gel and then transferred onto PVDF membranes (Millipore, Billerica, MA). After blocking membranes, they were incubated with appropriate dilutions (1:1000) of specific primary antibodies. The blots were incubated with HRP-conjugated secondary antibodies and visualized using ECL system (Thermo Fisher Scientific, Rochester, NY), Anti-mouse/rabbit second antibody for Western Blot was from Invitrogen.

### *In vivo* studies

Male 6- to 8-week old nude mice were used. 8 mice were injected subcutaneously with 1 × 10^6^ C4-2 cells pre-co-cultured with mast cells for 1 week, as a mixture with Matrigel, 1:1 and another8 mice were injected with 1 × 10^6^ C4-2 cells. After 2 weeks, the mice were treated with docetaxel (15 mg/kg, 2 times/week) for 3 weeks, then the mice were sacrificed. All animal studies were performed under the supervision and guidelines of the Xi'an Jiaotong University Animal Care and Use Committee.

### Histology and IHC staining

Mouse prostate tissues were fixed in 10% (v/v) formaldehyde in PBS, embedded in paraffin, and cut into 5 μm sections. Prostate sections were deparaffinized in xylene solution and rehydrated using gradient ethanol concentrations, and immunostaining was performed.

### Statistics

All statistical analyses were carried out with SPSS 19.0 (SPSS Inc, Chicago, IL). The data values were presented as the mean ± SEM. Differences in mean values between two groups were analyzed by two-tailed Student's *t* test, and the means of more than two groupswere compared with one way ANOVA. *p* ≤ 0.05 was considered statistically significant.

## SUPPLEMENTARY FIGURES


