# Correction: Infiltrating mast cells increase prostate cancer chemotherapy and radiotherapy resistances via modulation of p38/p53/p21 and ATM signals

**DOI:** 10.18632/oncotarget.27023

**Published:** 2019-06-11

**Authors:** Hongjun Xie, Chong Li, Qiang Dang, Luke S. Chang, Lei Li

**Affiliations:** ^1^ Chawnshang Chang Sex Hormone Research Center, Department of Urology, The First Affiliated Hospital, Xi’an Jiaotong University, Xi’an 710061, China; ^2^ CAS Key Laboratory of Infection and Immunity, Institute of Biophysics, Chinese Academy of Science, Beijing 100101, China; ^*^These authors have contributed equally to this work

**This article has been corrected:** Due to errors in figure preparation, the image of 22Rv1 displayed in Figure 1B is incorrect. The corrected figure is shown below. The authors declare that these corrections do not change the results or conclusions of this paper.

Original article: Oncotarget. 2016; 7:1341–1353. 1341-1353 . https://doi.org/10.18632/oncotarget.6372

**Figure 1:**
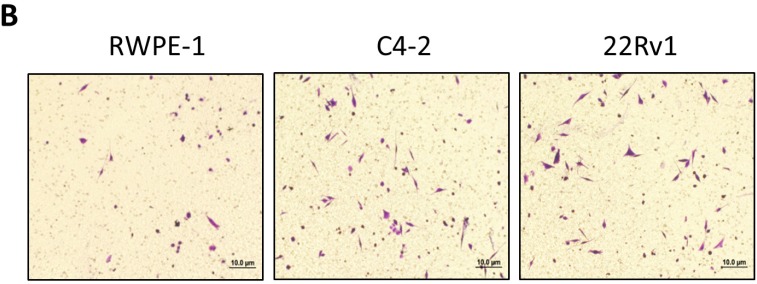
Prostate cancer recruits more mast cells than normal prostate. **B.** PCa cells promote mast cell migration. Mast cells (1 × 105) were added in the upper well, we placed non-malignant prostate RWPE-1 cell conditioned medium and PCa C4-2 and CWR22Rv1 (22Rv1) cells conditioned medium to do migration assay. The right panel is the quantitative data for migrated mast cells. Results were presented as the average values and represented as mean± SEM. *p < 0.05.

