# Supplementary material for: Infiltrating mast cells increase prostate cancer chemotherapy and radiotherapy resistances via modulation of p38/p53/p21 and ATM signals
Source: Oncotarget. 2015 Nov 16;7(2):1341–53. doi: 10.18632/oncotarget.6372 (PMC4811464; doi:10.18632/oncotarget.6372)
Supplement: Supplementary file 1 [file oncotarget-07-1341-s001.pdf]

SUPPLEMENTARY FIGURES

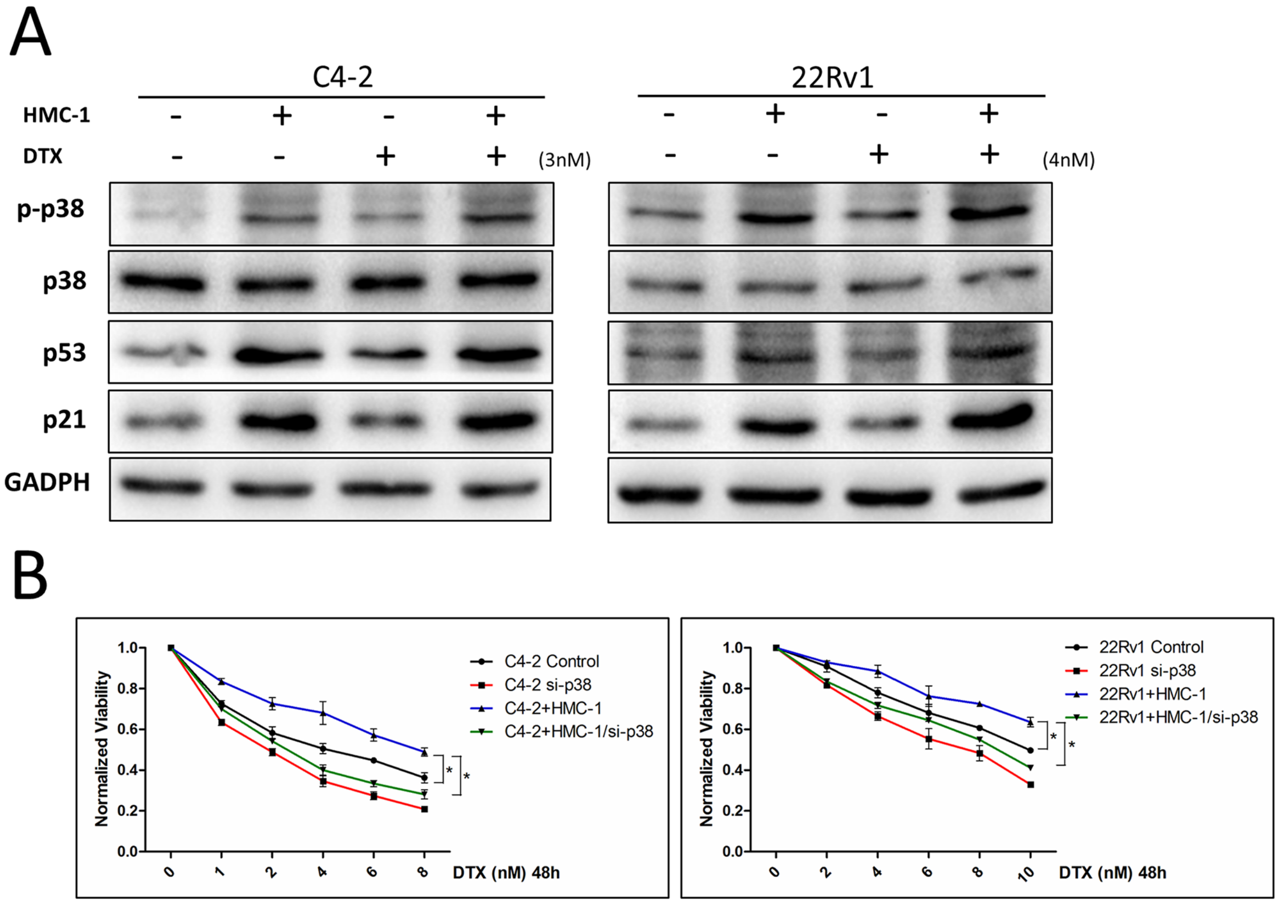

Supplementary Figure S1: Mechanism why recruited mast cells can alter PCa cell chemotherapy sensitivity. A. PCa C4-2 and CWR22Rv1(22Rv1) cells show increased expression of p-p38, p53 and p21 after co-culture with mast cells in the present of DTX. **B.** Targeting p38 with p38 si-RNA can interrupt mast cell-induced docetaxel resistance.

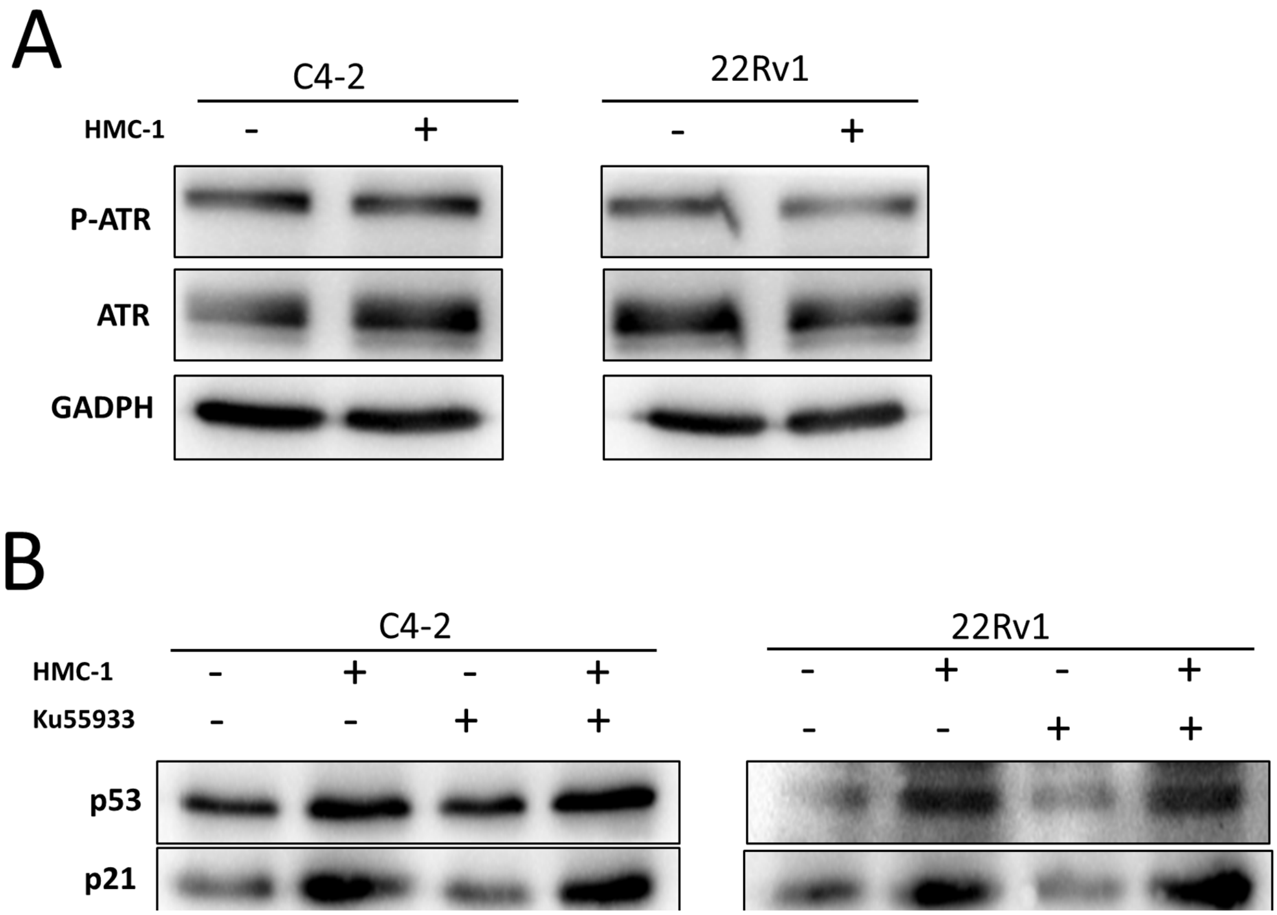

Supplementary Figure S2: Effect of mast cells and ATM kinase inhibitor on the down steam gene expression. A. Mast cells has no effect on phosphorylation of ATR in both C4-2 and CWR22Rv1 (22Rv1) cells. **B.** Expression of p-p38, p53 and p21 after co-culture with mast cells in the present of ATM kinase inhibitor.

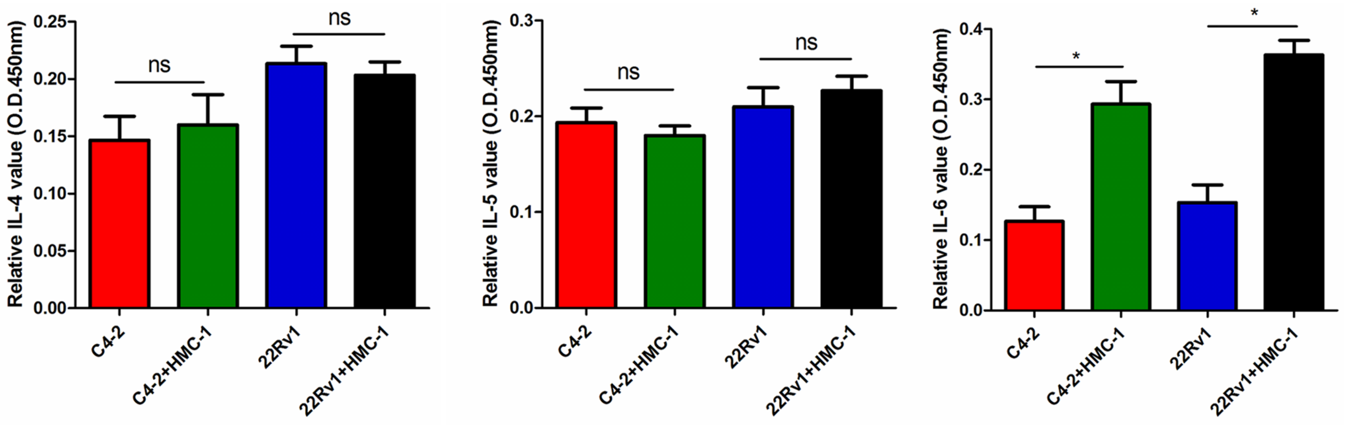

Supplementary Figure S3: Screen of mast cells secreted cytokines potentially affecting p38 signal. Detection of the IL-4, IL-5 and IL-6 expression in the medium of prostate cancer cells with or without co-culture with mast cells.
